# Retraction: Prader–Willi region non-protein coding RNA 1 suppressed gastric cancer growth as a competing endogenous RNA of miR-425-5p

**DOI:** 10.1042/CS-2017-1588_RET

**Published:** 2024-05-22

**Keywords:** gastric cancer, miR-425-5p, p53 signaling pathway, PTEN, PWRN1

This article is being retracted from *Clinical Science* at the request of the Editor-in-Chief and the Editorial Board following receipt of a notification from a reader, alerting the Editorial Board to multiple image segment duplications within the article, as well as similarities between Figure S2A and a figure in a separate paper by different authors. Specifically:

- A part of Figure 2E pCMV6 MGC-803 panel which appears in Figure 5B MGC-803 NC panel
- A part of Figure 2E pCMV-PWRN1 MGC-803 panel which appears in Figure 5B MGC-803 miR-425-5p-inhibitor panel
- A part of Figure 5B HGC-27 NC panel which appears in Figure 5B HGC-27 miR-425-5p inhibitor panel
- A part of Figure 5B HGC-27 miR-425-5p mimics panel which appears in Figure 5B HGC-27 miR-425-5p mimics+pCMV-PWRN1 panel
- A part of Figure 5B MGC-803 miR-425-5p mimics panel which appears in Figure 5B MGC-803 miR-425-5p mimics+pCMV-PWRN1 panel
- A part of Figure S2C MGC-803 pCMV-PWRN1 0h panel appears in Figure S2C MGC-803 pCMV-PWRN1 24h panel
- Similarities between Figure S2A MGC-803 pCMV6 panel and Figure 4C NC panel from Fu et al 2018 (DOI: 10.1007/s13311-018-0649-9).

The authors have been contacted with regards to the retraction and have not responded to the Journal's queries or the concerns raised. Given the extent of the issues raised, the Editorial Board stand by the decision to retract the article.

